# Bogazici university smartphone accelerometer sensor dataset

**DOI:** 10.1016/j.dib.2022.107833

**Published:** 2022-01-16

**Authors:** Erhan Davarcı, Emin Anarım

**Affiliations:** Bogazici University Electrical and Electronics Engineering, Istanbul, Turkey

**Keywords:** Smartphones, Motion sensors, Accelerometer, Behavioral biometrics, Age-group detection, Gender recognition, Authentication

## Abstract

Mobile devices especially smartphones have gained high popularity and become a part of daily life in recent years. Therefore, there are many studies that investigate users' interactions with smartphones and try to extract meaningful information from various inputs. Actually, the main motivation behind these studies is the behavioral differences of users in their interactions with smartphones. In these studies, motion sensors in devices such as accelerometer and gyroscope are widely used. Data obtained from motion sensors allows to detect information such as age-group, gender, activity type, identity of users. In this context, we develop an Android application that gathers accelerometer sensor data while users perform different activities. This application records all accelerometer data and touch event information generated while users are using their devices. Then, we perform two experiments and collect two different data using this application. In the first experiment, we collect data from 107 child users and 100 adult users to analyze the impact of different age-groups' behavior on sensor data. This dataset includes more than 11.000 taps data for child and adult users, in total. In the second experiment, data is collected from 60 female and 60 male users for different activities like sitting and walking. There are more than 6.000 taps data for sitting and walking scenarios separately in the second dataset. This dataset makes it possible to analyze the changes created by different gender and activity types in the sensor data. These data can be used for behavioral biometric analyses on smartphones such as user age-group and gender detection, user identification and authentication.

## Specifications Table

**Table:** 

Subject	Computer Science: Cryptography and Cybersecurity, Signal Processing, Information Systems
Specific subject area	Behavioral Biometrics and Smartphone Motion Sensors
Type of data	Table
How the data were acquired	The data were acquired by an Android application that collects accelerometer data while user playing a balloon popping game. When the application starts, a balloon through different colors and sizes appears on a random position of the touch screen. As a user pops the balloon by touching it on the screen, the next one is appeared with a random color, size and position. While the application is running, all accelerometer data and touch event information are saved to the internal memory of the smartphone. Popular Android smartphones like Samsung Galaxy and LG G4 were used during the experiment.
Data format	Raw
Description of data collection	Age-group data was collected from 107 child and 100 adult users. At least 50 tap events were obtained for each user. In gender data, data were collected from 60 female and 60 male users in different activities. Users were not given any guidance on holding and touching devices. Different smartphone models with different screen size were used and their sampling rate was 100 Hz.
Data source location	Institution: Bogazici University City/Town/Region: Istanbul Country: Turkey Latitude and longitude (and GPS coordinates, if possible) for collected samples/data: 41° 5′ 5.009″ N 29° 3′ 3.666″ E
Data accessibility	Repository name: Mendeley Data Data identification number: http://dx.doi.org/10.17632/djr93wwgj3.5 Direct URL to data: https://data.mendeley.com/datasets/djr93wwgj3/5
Related research article	E. Davarci, B. Soysal, I. Erguler, S. O. Aydin, O. Dincer and E. Anarim, ``Age group detection using smartphone motion sensors,'' 2017 25th European Signal Processing Conference (EUSIPCO), 2017, pp. 2201-2205, doi:10.23919/EUSIPCO.2017.8081600. E. Davarci and E. Anarim, ``Gender Detection with Smartphone Motion Sensors Using Convolutional Neural Networks,'' 2021 29th Signal Processing and Communications Applications Conference (SIU), 2021, pp. 1-4, doi:10.1109/SIU53274.2021.9478004. E. Davarci and E. Anarim, ``Hybrid Architecture for Gender Recognition Using Smartphone Motion Sensors,'' 2021 29th European Signal Processing Conference (EUSIPCO), 2021, pp. 801-805, doi:10.23919/EUSIPCO54536.2021.9616259.

## Value of the Data

• This data is useful to analyze and derive behavioral biometrics of smartphone users. By using this data, different data-driven models can be developed to analyze touching, holding or walking attributes of smartphone users. This data help researchers to train and evaluate their models for different user groups and scenarios.
• There is not much dataset on smartphone motion sensors. In addition, similar datasets have small amount of data from specific user groups. Since this dataset includes large amount of data from various user groups in different activities, it can be valuable students, engineers, and researchers studying behavioral biometrics on smartphones.
• With this data set, models that predict information such as user age, gender and activity can be developed. For this purpose, this dataset has been already used in some academic studies [1], [2], [3]. Application developers can develop new techniques by using this data to enrich the user experience on smartphones like automatically customizing screen or applications and providing more relevant search results.
• Security researchers can also use this dataset to develop new user identification and authentication mechanism [4], [5], [6]. Information about users derived from sensor data can be used as a secondary layer of authentication. By using this data researchers can test the effects of different user groups on security models and the success rates of the models.
• In the literature, there are some studies trying to extract information about user input on smartphones using motion sensors [7], [8], [9]. This dataset provides a general understanding about how different user behaviors are reflected in motion sensors. Therefore, these studies can benefit this dataset and improve their techniques.

## 1 Data Description

This paper consists of a dataset about accelerometer sensor data of smartphones. The data was gathered during users interact with touchscreen of a smartphone. In order to collect data, we performed two experiments and collect two different datasets, namely age-group and gender dataset. Therefore, the dataset contains two main folders, age-group dataset and gender dataset folders.

In the first experiment, data from 107 child users and 100 adult users were collected with popular Android smartphones. This dataset consists of 5268 tap events from adult users and 6088 tap events from child users, shown in Table 1.

**Table 1:** Samples in the age-group dataset.

User type	# of users	# of tap events
Adult	100	5268
Child	107	6088
Total	207	11356

Age-group dataset contains two subfolders, namely Adult and Child. The first one includes data from adult users. Since we collected data from 100 adult users, there are 100 text files in this folder and each file corresponds to data from one user and approximately includes 45 s accelerometer data of that user. Similarly, the second folder includes data from children. Note that, text file names include only user id and age information about users. For example, filename ``UserID1001-16-Accelerometer.txt'' means that this accelerometer data belongs to a user with user ID 1001 and age 16. Since we did not record other information about users like name, identity etc., there is no privacy issue about the data.

In the second experiment, we collected data from 60 female (average age = 35.6, min = 18, max = 57) and 60 male (average age = 30.3, min = 17, max = 57) users. We put the collected data under the gender dataset main folder. Gender dataset include gender and activity information in addition to the user's age. There are two different activities in this dataset as sitting and walking. Therefore, gender dataset contains two subfolders, namely female and male. Each subfolder also contains two separate folders, sitting and walking. Female folder includes data from female user only and sitting subfolder includes data for sitting scenario whereas walking subfolder includes data for walking one. The same structure applies to the male folder.

Gender dataset consists of 6247 tap events from female users and 6414 tap events from male users, shown in Table 2. In gender dataset, text file names include user id, gender, age and activity information about users. For example, filename ``UserID2001-F-24-Sitting-Accelerometer.txt'' means that this accelerometer data belongs to a user with user ID 2001, a 24-year-old female user and user is sitting while the data is being acquired.

**Table 2:** Samples in the gender dataset.

User type	# of users	Activity	# of tap events
Female	60	Sitting	3116
		Walking	3131
Male	60	Sitting	3190
		Walking	3224
Total	120	Both	12661

Text files in the both Age-Group and Gender dataset include raw data. Raw data means that text file includes five columns. The first column is the timestamp information that is the time in nanosecond at which the sensor event occurred. The next three columns are accelerometer readings in x, y and z directions, respectively. The last column gives information about touch events like *Action_Down, Action_Move, Action_Up.*

Columns in each text file are as follows:

• **First column:** Timestamp at which the sensor data are acquired in nanosecond (*ns*).
• **Second column:** Value of the x-axis of accelerometer readings (*m/s^2^*).
• **Third column:** Value of the y-axis of accelerometer readings (*m/s^2^*).
• **Fourth column:** Value of the z-axis of accelerometer readings (*m/s^2^*).
• **Fifth column:** Touch event information such as *Action_Down (1), Action_Move (2), Action_Up (-1).*

An example of five consecutive sensor readings in a text file is illustrated in Table 3.

**Table 3:** An example of five consecutive sensor readings in a text file.

Timestamp	Accelerometer_x	Accelerometer_y	Accelerometer_z	Touch State
651998837000	−2.3517046	5.281609	14.192827	1
652009950000	−1.9087774	5.7137623	12.996924	2
652018914000	−2.4037786	5.129577	9.251195	2
652030558000	−2.6072857	4.584896	5.5030727	2
652038959000	−2.296638	4.5675383	3.364452	−1

Touch event information in the recorded data can take value of one of three touch states: *Action_Down, Action_Move, Action_Up*. We defined these three touch states in application software as follows: When user touches the screen, it is an *Action_Down* state and touch state is 1. *Action_Up* means user removes her finger from touchscreen i.e., touching has finished, in this case touch state is -1. The state between *Action_Down* and *Action_Up* is *Action_Move* phase and touch state is 2. Note that default value of touch state is 0, i.e., touch state is 0 until the user first touches the screen. By using this touch state information, we can easily determine tap locations in accelerometer readings.

**Fig. 1:**
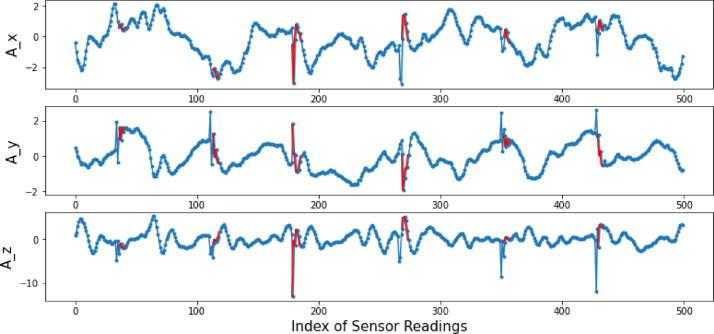
Example raw accelerometer sensor readings.

An example of raw accelerometer sensor readings in x, y and z directions is shown in Fig. 1. By the help of touch state information, tap locations in the plot are highlighted with red color.

## 2 Experimental Design, Materials and Methods

We developed a mobile application using the Android Studio development environment to collect data and we named this application as *BalloonLogger* [10]. This developed application logs the accelerometer data on the x, y and z axes resulting from the user's movements or interactions with the touchscreen. We chose the Android platform and implemented our experimental application on Android-based smartphones because:

a) It is an open-source platform and extensively used in smartphones of different vendors.
b) Android makes it possible installation of applications from sources other than Google Play Store. Therefore, installing process of a third-party app (like our *BalloonLogger*) does not require rooting the device.
c) Android apps don't need permission to use the accelerometer sensor and record its data. Only apps that need to receive sensor data at a higher rate like 200 Hz and above need permission [11]. Since our application has 100 Hz sampling frequency and we do not collect any user-specific data, there is no concern about permissions in our study.

BalloonLogger is actually a simple game application and works like in this way: User information such as age and gender are entered and the application is started by pressing the *START* button. Throughout the game, 50 balloons of different colors and sizes appear in order in different parts of the screen and the user is asked to destroy the balloons by touching the screen. When a balloon is destroyed, the next one will appear with a random size and position. The game is over after all the balloons are gone. In order to keep the kids entertained during the test, we also adopt sound effects and scoring to *BalloonLogger*. All accelerometer data and touch event information are recorded on the smartphone while *BalloonLogger* is running. Then, this data is transferred to PC for further analysis.

In the analysis of sensor readings, the sampling rate of the sensor data is critical. Low sampling rate cannot give enough information to process sensor data. The sampling rate depends on the smartphone used and sensor delay assigned in application code. Actually, different sampling frequencies can be assigned to read data in Android. Therefore, we set the sampling rate of the application to 100 Hz in smartphones used in these experiments.

By using this application, we performed two different experiments and collected data from users. We used 5 different smartphones like Samsung Galaxy S3, Galaxy S4, Galaxy S5, LG G3 and G4 as test device during the both experiments. Before the experiments, the developed application, *BalloonLogger*, was packaged as apk file and distributed to these test devices via USB and installed. In the experiments, one of these test devices was given to the users at random, the users did not use their own devices. Furthermore, users were not guided about how they hold or touch the device while playing *BalloonLogger.*

In the first experiment, we collected data from 107 children aged 4 to 11, and 100 adult users aged 16 to 55. In this experiment, all users were in a sitting position while using the application. In the second experiment, data from 60 female users aged 18 to 57 and 60 male users aged 17 to 57 were collected. We collected data from users in both sitting and walking scenarios in the second experiment. In sitting scenario, users are playing *BalloonLogger* with the smartphone in their hands while in a sitting position. Whereas, users are walking and playing games in their smartphone hands in walking scenario.

For adult data collection we asked from our friends, colleagues and families to play our test game and child data was also collected from the children of these individuals. Data collection was done on a completely voluntary basis, and data were collected only from users who wanted to participate voluntarily. Users who wanted to participate in the study were expected to have previous smartphone usage experience, this was the only inclusion criteria and no exclusion criteria applied. Data collection was carried out in environments such as workplace, home, shopping mall, where the participants do their daily work. For users under the age of 18, after giving detailed information to their parents and obtaining their consent, data collection was carried out in the presence of their parents. In addition, data collection for all users took place under our control and with us. Therefore, user information such as age and gender were supervised, for children this information was also verified from their parents.

## Ethics Statement

The procedure of the methodology used in this study was approved by the Institutional Review Board for Research with Human Subjects of Bogazici University with the approval number 2022/02. The data collection procedure and all of the interventions in this research fully meet the 1964 Declaration of Helsinki. All the users participated voluntarily in this experiment, they were informed about the data acquisition process and their informed consent obtained. All participants were also informed about the data distribution and sharing for further research. For users under the age of 18, data collection was carried out in the presence of their parents after detailed information was given to their parents and their consent was obtained. In addition, the user's data are fully anonymized.

## CRediT authorship contribution statement

**Erhan Davarcı:** Software, Investigation, Methodology, Conceptualization. **Emin Anarım:** Supervision.

## Declaration of Competing Interest

The authors declare that they have no known competing for financial interests or personal relationships that could have appeared to influence the work reported in this paper.

## Appendix Supplementary materials

Image, application 1
