# Supplementary material for: Bogazici university smartphone accelerometer sensor dataset
Source: Data Brief. 2022 Jan 16;40:107833. doi: 10.1016/j.dib.2022.107833 (PMC8792433; doi:10.1016/j.dib.2022.107833)
Supplement: Supplementary file 1 [file mmc1.pdf]

T.C.
BOĞAZİÇİ ÜNİVERSİTESİ REKTÖRLÜĞÜ
Fen Bilimleri ve Mühendislik Alanları İnsan Araştırmaları Etik Kurulu
(FMİNAREK)

Sayı : E-84391427-050.01.04-46187 05.01.2022
Konu : 2022/02 Kayıt no'lu başvurunuz hakkında

Sayın Prof. Dr. Emin ANARIM
Elektrik Elektronik Mühendisliği Bölüm Başkanlığı - Öğretim Üyesi

"Akıllı Cihazlardaki Kullanıcı Davranışlarının Dokunmatik Ekran ve Sensör Verileri Aracılığı ile Analiz Edilmesi" başlıklı projeniz ile Boğaziçi Üniversitesi Fen Bilimleri ve Mühendislik Alanları İnsan Araştırmaları Etik Kurulu (FMİNAREK)'e yaptığınız 2022/02 kayıt numaralı başvuru 03.01.2022 tarihli ve 2022/01 No.lu kurul toplantısında incelenerek etik onay verilmesi uygun bulunmuştur. Bu karar tüm üyelerin toplantıya on-line olarak katılımıyla ve oybirliği ile alınmıştır.

COVID-19 önlemleri nedeniyle üyelerden ıslak imza alınamadığından bu onam mektubu tüm üyeler adına Komisyon Başkanı tarafından e-imzalanmıştır.

Saygılarımızla bilginize sunarız.

Prof. Dr. Tınaz EKİM AŞICI
Başkan

Bu belge, güvenli elektronik İmza ile imzalanmıştır.
Doğrulama Kodu :BSV3CT14BZ Pin Kodu :40172 Belge Takip Adresi : https://turkiye.gov.tr/ebd?eK=4787&eD=BSV3CT14BZ&eS=46187

34342 Bebek-İstanbul
Telefon No:0212 287 17 53 Faks No:0212 265 70 06
İnternet Adresi:www.boun.edu.tr
Kep Adresi:bogaziciuniversitesi@hs01.kep.tr
Bilgi için: Nurşen MUNAR
Unvan: Mühendis

Bu belge, güvenli elektronik imza ile imzalanmıştır.
